# Multimodal and Multiscale Deep Neural Networks for the Early Diagnosis of Alzheimer’s Disease using structural MR and FDG-PET images

**DOI:** 10.1038/s41598-018-22871-z

**Published:** 2018-04-09

**Authors:** Donghuan Lu, Karteek Popuri, Gavin Weiguang Ding, Rakesh Balachandar, Mirza Faisal Beg, Alzheimer’s Disease Neuroimaging Initiative, Michael Weiner, Paul Aisen, Ronald Petersen, Cliford Jack, William Jagust, John Trojanowki, Arthur Toga, Laurel Beckett, Robert Green, Andrew Saykin, John Morris, Leslie Shaw, Jefrey Kaye, Joseph Quinn, Lisa Silbert, Betty Lind, Raina Carter, Sara Dolen, Lon Schneider, Sonia Pawluczyk, Mauricio Beccera, Liberty Teodoro, Bryan Spann, James Brewer, Helen Vanderswag, Adam Fleisher, Judith Heidebrink, Joanne Lord, Sara Mason, Colleen Albers, David Knopman, Kris Johnson, Rachelle Doody, Javier Villanueva-Meyer, Munir Chowdhury, Susan Rountree, Mimi Dang, Yaakov Stern, Lawrence Honig, Karen Bell, Beau Ances, Maria Carroll, Mary Creech, Erin Franklin, Mark Mintun, Stacy Schneider, Angela Oliver, Daniel Marson, Randall Grifth, David Clark, David Geldmacher, John Brockington, Erik Roberson, Marissa Natelson Love, Hillel Grossman, Efe Mitsis, Raj Shah, Leyla deToledo-Morrell, Ranjan Duara, Daniel Varon, Maria Greig, Peggy Roberts, Marilyn Albert, Chiadi Onyike, Daniel D’Agostino, Stephanie Kielb, James Galvin, Brittany Cerbone, Christina Michel, Dana Pogorelec, Henry Rusinek, Mony de Leon, Lidia Glodzik, Susan De Santi, P. Doraiswamy, Jefrey Petrella, Salvador Borges-Neto, Terence Wong, Edward Coleman, Charles Smith, Greg Jicha, Peter Hardy, Partha Sinha, Elizabeth Oates, Gary Conrad, Anton Porsteinsson, Bonnie Goldstein, Kim Martin, Kelly Makino, M. Ismail, Connie Brand, Ruth Mulnard, Gaby Thai, Catherine Mc-Adams-Ortiz, Kyle Womack, Dana Mathews, Mary Quiceno, Allan Levey, James Lah, Janet Cellar, Jefrey Burns, Russell Swerdlow, William Brooks, Liana Apostolova, Kathleen Tingus, Ellen Woo, Daniel Silverman, Po Lu, George Bartzokis, Neill Graf-Radford, Francine Parftt, Tracy Kendall, Heather Johnson, Martin Farlow, Ann Marie Hake, Brandy Matthews, Jared Brosch, Scott Herring, Cynthia Hunt, Christopher Dyck, Richard Carson, Martha MacAvoy, Pradeep Varma, Howard Chertkow, Howard Bergman, Chris Hosein, Sandra Black, Bojana Stefanovic, Curtis Caldwell, Ging-Yuek Robin Hsiung, Howard Feldman, Benita Mudge, Michele Assaly, Elizabeth Finger, Stephen Pasternack, Irina Rachisky, Dick Trost, Andrew Kertesz, Charles Bernick, Donna Munic, Marek-Marsel Mesulam, Kristine Lipowski, Sandra Weintraub, Borna Bonakdarpour, Diana Kerwin, Chuang-Kuo Wu, Nancy Johnson, Carl Sadowsky, Teresa Villena, Raymond Scott Turner, Kathleen Johnson, Brigid Reynolds, Reisa Sperling, Keith Johnson, Gad Marshall, Jerome Yesavage, Joy Taylor, Barton Lane, Allyson Rosen, Jared Tinklenberg, Marwan Sabbagh, Christine Belden, Sandra Jacobson, Sherye Sirrel, Neil Kowall, Ronald Killiany, Andrew Budson, Alexander Norbash, Patricia Lynn Johnson, Thomas Obisesan, Saba Wolday, Joanne Allard, Alan Lerner, Paula Ogrocki, Curtis Tatsuoka, Parianne Fatica, Evan Fletcher, Pauline Maillard, John Olichney, Charles DeCarli, Owen Carmichael, Smita Kittur, Michael Borrie, T.-Y. Lee, Rob Bartha, Sterling Johnson, Sanjay Asthana, Cynthia Carlsson, Steven Potkin, Adrian Preda, Dana Nguyen, Pierre Tariot, Anna Burke, Nadira Trncic, Stephanie Reeder, Vernice Bates, Horacio Capote, Michelle Rainka, Douglas Scharre, Maria Kataki, Anahita Adeli, Earl Zimmerman, Dzintra Celmins, Alice Brown, Godfrey Pearlson, Karen Blank, Karen Anderson, Laura Flashman, Marc Seltzer, Mary Hynes, Robert Santulli, Kaycee Sink, Leslie Gordineer, Jef Williamson, Pradeep Garg, Franklin Watkins, Brian Ott, Henry Querfurth, Geofrey Tremont, Stephen Salloway, Paul Malloy, Stephen Correia, Howard Rosen, Bruce Miller, David Perry, Jacobo Mintzer, Kenneth Spicer, David Bachman, Nunzio Pomara, Raymundo Hernando, Antero Sarrael, Norman Relkin, Gloria Chaing, Michael Lin, Lisa Ravdin, Amanda Smith, Balebail Ashok Raj, Kristin Fargher

**Affiliations:** 1 School of Engineering Science, Simon Fraser University, Burnaby, V5A 1S6 Canada; 2 Magnetic Resonance Unit at the VA Medical Center and Radiology, Medicine, Psychiatry and Neurology, University of California, San Francisco, USA; 3 San Diego School of Medicine, University of California, California, USA; 4 Mayo Clinic, Minnesota, USA; 5 Mayo Clinic, Rochester, USA; 6 University of California, Berkeley, USA; 7 University of Pennsylvania, Pennsylvania, USA; 8 University of Southern California, California, USA; 9 University of California, Davis, California, USA; 10 MPH Brigham and Women’s Hospital/Harvard Medical School, Massachusetts, USA; 11 Indiana University, Indiana, USA; 12 Washington University St. Louis, Missouri, USA; 13 Oregon Health and Science University, Oregon, USA; 14 University of California–San Diego, California, USA; 15 University of Michigan, Michigan, USA; 16 Baylor College of Medicine, Houston, State of Texas USA; 17 Columbia University Medical Center, South Carolina, USA; 18 University of Alabama, Birmingham, Alabama USA; 19 Mount Sinai School of Medicine, New York, USA; 20 Rush University Medical Center, Rush University, Illinois, USA; 21 Wien Center, Florida, USA; 22 Johns Hopkins University, Maryland, USA; 23 New York University, NY, USA; 24 Duke University Medical Center, North Carolina, USA; 25 University of Kentucky, Kentucky, USA; 26 University of Rochester Medical Center, NY, USA; 27 University of California, Irvine, California, USA; 28 University of Texas Southwestern Medical School, Texas, USA; 29 Emory University, Georgia, USA; 30 University of Kansas, Medical Center, Kansas, USA; 31 University of California, Los Angeles, California, USA; 32 Mayo Clinic, Jacksonville, USA; 33 Yale University School of Medicine, Connecticut, USA; 34 McGill University, Montreal-Jewish General Hospital, Montreal, Canada; 35 Sunnybrook Health Sciences, Ontario, USA; 36 U.B.C. Clinic for AD & Related Disorders, Vancouver, BC Canada; 37 Cognitive Neurology - St. Joseph’s, Ontario, USA; 38 Cleveland Clinic Lou Ruvo Center for Brain Health, Ohio, USA; 39 Northwestern University, San Francisco, USA; 40 Premiere Research Inst (Palm Beach Neurology), west Palm Beach, USA; 41 Georgetown University Medical Center, Washington DC, USA; 42 Brigham and Women’s Hospital, Massachusetts, USA; 43 Stanford University, California, USA; 44 Banner Sun Health Research Institute, Sun City, AZ 85351 USA; 45 Boston University, Massachusetts, USA; 46 Howard University, Washington DC, USA; 47 Case Western Reserve University, Ohio, USA; 48 University of California, Davis – Sacramento, California, USA; 49 Neurological Care of CNY, Liverpool, NY 13088 USA; 50 Parkwood Hospital, Pennsylvania, USA; 51 University of Wisconsin, Wisconsin, USA; 52 University of California, Irvine – BIC, California, USA; 53 Banner Alzheimer’s Institute, Phoenix, 85006 Arizona USA; 54 Dent Neurologic Institute, NY, USA; 55 Ohio State University, Ohio, USA; 56 Albany Medical College, NY, USA; 57 Hartford Hospital, Olin Neuropsychiatry Research Center, Connecticut, USA; 58 Dartmouth-Hitchcock Medical Center, New Hampshire, USA; 59 Wake Forest University Health Sciences, North Carolina, USA; 60 Rhode Island Hospital, state of Rhode Island, Providence, RI 02903 USA; 61 Butler Hospital, Providence, Rhode Island USA; 62 University of California, San Francisco, USA; 63 Medical University South Carolina, Charleston, SC 29425 USA; 64 Nathan Kline Institute, Orangeburg, New York, USA; 65 Cornell University, Ithaca, New York, USA; 66 USF Health Byrd Alzheimer’s Institute, University of South Florida, Tampa, FL 33613 USA

## Abstract

Alzheimer’s Disease (AD) is a progressive neurodegenerative disease where biomarkers for disease based on pathophysiology may be able to provide objective measures for disease diagnosis and staging. Neuroimaging scans acquired from MRI and metabolism images obtained by FDG-PET provide in-vivo measurements of structure and function (glucose metabolism) in a living brain. It is hypothesized that combining multiple different image modalities providing complementary information could help improve early diagnosis of AD. In this paper, we propose a novel deep-learning-based framework to discriminate individuals with AD utilizing a multimodal and multiscale deep neural network. Our method delivers 82.4% accuracy in identifying the individuals with mild cognitive impairment (MCI) who will convert to AD at 3 years prior to conversion (86.4% combined accuracy for conversion within 1–3 years), a 94.23% sensitivity in classifying individuals with clinical diagnosis of probable AD, and a 86.3% specificity in classifying non-demented controls improving upon results in published literature.

## Introduction

Alzheimer’s disease (AD), the most common dementia, affecting 1 out of 9 people over the age of 65 years^1^. Alzheimer’s diseases involves progressive cognitive impairment, commonly associated with early memory loss, requiring assistance for activities of self care during advanced stages. Alzheimer’s is posited to evolve through a prodromal stage which is commonly referred to as the mild cognitive impairment (MCI) stage and 10–15% of individuals with MCI, progress to AD^2^ each year. With improved life expectancy, it is estimated that about 1.2% of global population will develop Alzheimer’s disease by 2046^3^ thereby affecting millions of individuals directly, as well as many more indirectly through the effects on their families and caregivers. There is an urgent need to develop biomarkers that can identify the changes in a living brain due to the pathophysiology of AD providing numerical staging scores, as well as identifying syndromal stages.

Neuroimaging modalities such as magnetic resonance imaging (MRI)^4^ and fluorodeoxyglucose positron emission tomography (FDG-PET)^5^ have been previously used to develop such pathophysiology-based biomarkers for diagnosis of AD, specially targeting the prodromal stage of AD, where the pathology has begun but the clinical symptoms have not yet manifested. Structural MRI provides measures of brain gray matter, white matter and CSF compartments enabling the quantification of volumes, cortical thickness and shape of various brain regions and utilize these in developing classifiers for AD^6–13^. FDG-PET provides measures of the resting state glucose metabolism^14^, reflecting the functional activity of the underlying tissue^5^ that has also been utilized for AD biomarker development^15–17^. Other published approaches have utilized a combination of modalities for developing neuroimaging AD biomarkers^4,18–24^.

Recent advances in deep neural network approaches for developing classifiers have delivered astounding performance for many recognition tasks^25^. The application of deep neural networks in recognition of AD has also attracted application for AD^26–28^. By applying deep neural network to extract features, such as stacked autoencoder (SAE) or Deep Boltzmann Machine (DBM), these approaches outperform other popular traditional machine learning methods, e.g., support vector machine (SVM) and random forest techniques. A major problem of deep neural network’s application in AD diagnosis is that only a small amount of training data is available for learning discriminative patterns in very high dimensional feature spaces. Another issue is that the scale at which the discriminative signal resides is not a-priori known hence dimensionality reduction techniques need to be sensitive to multiple scales to increase the chances of extracting the discriminative signal.

In this paper, we are proposing a novel approach for combining multimodal information from both MRI and FDG-PET images at multiple scales within a deep neural network framework. Our proposed multiscale approach extracts features at coarse-to-fine structural scales^29,30^. This is achieved by segmenting the structural image into cortical and subcortical gray-matter compartments, and further subdividing each into patches of a hierarchical size, and extract features from each-sized patch^26–28^ by averaging within the patch and use these multi-scale features taken from multiple modalities into a deep learning framework. Unlike the simple approach of down sampling, which could lead to the loss of discriminative information, our multi-scale approach preserves the structural and metabolism information at multiple scales and may potentially improve the classification accuracy for this diagnostic task^31^. To validate our proposed novel methodology, we performed cross validation experiments with all available ADNI data (subjects that include both a T1-structural MRI and an FDG-PET metabolism image). A comprehensive set of results of these experiments for the detection of controls and MCI that convert to AD as a function of years to conversion, as well as classification of controls, and AD subjects are presented for each modality separately and in combination, and compared to existing methods available in literature demonstrating superiority of the deep neural network framework in AD diagnosis and prognosis.

## Methods

There are two major steps in the proposed framework: (1)image preprocessing: segment both MRI and FDG-PET images, subdivide the gray-matter segmentation into patches of a range of sizes, and extract features from each-sized patch; and, (2)classification: train a deep neural network to learn the patterns that discriminate AD individuals, and then use for individual classification.

### Materials

Data used in the preparation of this article were obtained from the Alzheimer’s Disease Neuroimaging Initiative (ADNI) database (adni.loni.usc.edu). The ADNI was launched in 2003 as a public-private partnership, led by Principal Investigator Michael W. Weiner, MD. The primary goal of ADNI has been to test whether serial MRI, PET, other biological markers, and clinical and neuropsychological assessment can be combined to measure the progression of mild cognitive impairment (MCI) and early Alzheimer’s disease (AD).

For a comprehensive validation of the proposed method, it is emphasized that all the available ADNI subjects (N = 1242) with both a T1-weighted MRI scan and FDG-PET image at the time of preparation of this manuscript were used in this study. These subjects were categorized into 5 groups based on the individual’s clinical diagnosis at baseline and future-timepoints. 1) Stable Normal controls (sNC): 360 subjects diagnosed to be NC at baseline and remained NC at the time of preparation of this manuscript. 2) Stable MCI (sMCI): 409 subjects diagnosed to be MCI at all time points (at least for 2 years). 3) Progressive NC (pNC): 18 subjects evaluated to be NC at baseline visit but progressed to clinical diagnosis of probable AD at the time of preparation of this manuscript. 4) Progressive MCI (pMCI): 217 subjects evaluated to be MCI at baseline visit and progressed to a clinical diagnosis of probable AD at some point in the future (data available for upto 8 years prior to conversion for some individuals). 5) Stable Alzheimer’s disease (sAD): 238 subjects with a clinical diagnoses of probably AD. Subjects showing improvement in their clinical diagnosis during their follow up, i.e. those clinically diagnosed as MCI but reverted to NC or those clinically diagnosed as probable AD but reverted to MCI were excluded from the proposed study because of the potential uncertainty of clinical misdiagnosis considering AD is an irreversible form of dementia^1^. The progressive controls and progressive MCI subjects have some neuroimaging timepoints with a clinical diagnosis of probable AD. Hence, the subset of images from pNC and pMCI subjects that have a clinical diagnosis of probable AD will be identified as part of the sAD group for assessment of classifier accuracy, while the remaining images before the conversion to AD will be assessed as part of the pNC and pMCI groups. Demographic and clinical information of the subjects are shown in Table 1. Numbers in brackets are the number of male and female subjects in the second row, while in the rest 3 rows the two number represent the minimum and maximum value of age, education year and MMSE (Mini–Mental State Examination) score. In total, there are 2402 FDG-PET scans and 2402 MRI images including all the longitudinal time-points. Detailed descriptions of the ADNI subject cohorts, image acquisition protocols procedures and post-acquisition preprocessing procedures can be found at http://www.adni-info.org.

**Table 1:** Subject Demographics.

	sNC	sMCI	pNC	pMCI	sAD
Count (Male/Female)					
Number of Subjects	360 (167/193)	409 (239/170)	18 (11/7)	217 (126/91)	238 (141/97)
Number of Images	753 (399/354)	409 (239/170)	74 (51/23)	702 (422/280)	464 (270/194)
Mean (min-max)					
Age in years	73.4 (60–94)	74 (56–91)	77 (68–84)	74 (55–89)	75 (55–90)
Education in years	16.5 (6–20)	15.8 (7–20)	15.7 (12–20)	16.0 (8–20)	15.3 (4–20)
MMSE score	29.1 (24–30)	28.0 (22–30)	29.4 (27–30)	26.5 (9–30)	23.2 (18–27)

Except for the count where the numbers in brackets are (male/female), age (years), education (years) and MMSE score are displayed in the mean (min-max) format.

### Image Processing

**Figure 1:**
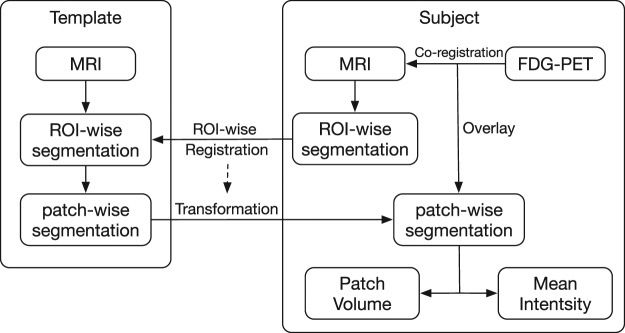
Flowchart of extracting patch-wise features from MRI scans and FDG-PET images. Each FreeSurfer ROI was segmented into “patches” through registration to a patch-segmented template. Patch-based volume and mean intensity of FDG-PET were extracted as features to represent each patch.

Unlike typical image recognition problems where deep learning has shown to be effective, our data set, although very large in a neuroimaging context, is relatively smaller. Hence directly using this smaller database of images to train the deep neural network is unlikely to deliver high classification accuracy. However, contrary to typical image recognition tasks, where the database of images contains large heterogeneity, the images in this database are all human brain images acquired with similar pose and scale which show relatively much less heterogeneity in comparison. Therefore we applied the following processing steps to extract patch-wise features as shown in Fig. 1: FreeSurfer 5.3^32^ was used to segment each T1 structural MRI image into gray matter and white matter followed by subdivision of the gray matter into 87 anatomical regions of interest (ROI). The FreeSurfer segmentation were quality controlled by an expert neuroanatomist and any errors noted were manually corrected. Then, a T1 MRI image was chosen as the template. Each ROI of this template was further subdivided into smaller regions of varying sizes, denoted here as “patches”. The voxels in each ROI were clustered into patches through *k*-means clustering based on Euclidean distance of their spatial coordinates^33^, i.e. voxels spatially close to each other would belong to the same patch. Given that the size of FreeSurfer ROIs were different, we predefined the number of voxels in each patch instead of fixing the number of patches in each ROI to keep uniform patch size density (patches in ROI/voxels in ROI) across the brain leading to signal aggregation at the same scale among the different ROIs. In this study, the size of patches was predefined to be 500, 1000 and 2000 voxels. Using these sizes, the number of patches in total across the brain gray matter ROIs segmented by FreeSurfer was found to be 1488, 705 and 343, respectively. The patch size chosen were designed to keep enough detailed information as well as avoiding too large feature dimension considering the limited number of available data samples. Subsequently, each ROI of the standard template MRI was registered to the same ROI of every target image via a high-dimensional non-rigid registration method (LDDMM^34^). The registration maps were then applied to the patch-wise segmentation of the standard template. This transformed the template patch segmentation into each target MRI space so the target images were subdivided into the same number of patches for their FreeSurfer ROIs. It is also worth mentioning that after the transformation, the size of a template patch in different images is not the same due to non-rigid registration encoding local expansion/contraction and hence is one of the features used to represent the regional information of a given structural brain scan. Then, for each target subject, the FDG-PET image of the subject was co-registered to its skull-stripped T1 MRI scan with a rigid transformation using FSL-FLIRT program^35^ based on normalized mutual information. The degrees of freedom (DOF) was set as 12 and Normalized correlation was used as cost function. The mean intensity in the brainstem region of the FDG-PET image was the chosen reference to normalize the voxel intensities in that individual brain metabolism image, because brainstem region was most unlikely to be affected by AD. The mean intensity of each patch was used to form the feature vector representing the metabolism activity, and the volume of each patch was used to represent the brain structure.

### Multimodal and Multiscale Deep Neural Network

**Figure 2:**
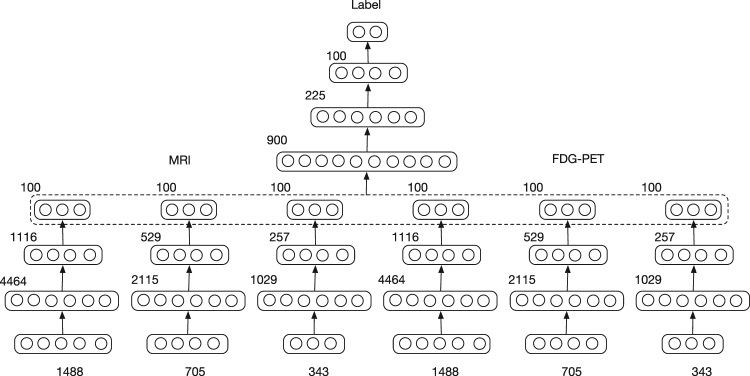
Multimodal and Multiscale Deep Neural Network. The input feature dimension (number of patches) extracted from different scales is 1488, 705 and 343. For each layer, its number of nodes is shown on the top left of the layer representation. For each scale of each image modality, its patch-wise measures were fed to a single DNN. The features from these 6 DNNs were fused by another DNN to generate the final probability score for each of the two classes being discriminated. Of the two classes, the class being the one with the highest probability (effectively a threshold of 0.5 for probability) is the assigned final classification. The probability output of the DNN can be interpreted as a staging score, with extreme value of 0 representing the highest probability of belonging to the sNC class, and extreme value of 1 representing the highest probability of belonging to the AD class.

With the features extracted from MRI and FDG-PET images, we trained a Multimodal and Multiscale Deep Neural Network (MMDNN) to perform the classification. As shown in Fig. 2, the network consists of two parts. The first part consisted of 6 independent deep neural networks (DNNs) corresponding to each scale of a single modality. The second part was another DNN used to fuse the features extracted from these 6 DNNs. The input data of this DNN was the concatenated latent representation learned from each single DNN. The DNNs in the two parts shared the same structure. For each DNN, the number of nodes for each hidden layer were set as 3*N*, $\tfrac{3}{4}N$ and 100 respectively, where *N* denotes the dimension of input feature vector. The number of nodes was chosen to explore all possible hidden correlation across features from different patches in the first layer and gradually reduce the number of features in the following layers to avoid over-fitting. We trained each DNN with two steps, unsupervised pre-training and supervised fine-tuning, respectively. Then all the parameters of MMDNN were tuned together. The trained DNN output is a probability value for each class, the final classification is to the label with the highest probability. The probability value can also be interpreted as a disease staging score, with extreme value of 0 representing the highest probability of belonging to the sNC class, and extreme value of 1 representing the highest probability of belonging to the AD class.

#### Unsupervised Pre-training

For the unsupervised pre-training step, each DNN was trained as a stacked-autoencoder (SAE). Autoencoder is an artificial neural network used for unsupervised learning of non-linear hidden patterns from input data. It consists of three layers, input layer, hidden layer and output layer, for which two nearby layers are fully-connected. Three functions are used to define an autoencoder, encoding function, decoding function and loss function. In this study, encoding function is defined as: *y* = *s* (*W*_1_*x* + *b*_1_), where *x* is the input data, *y* is the latent representation, *W*_1_ is the weight matrix, *b*_1_ is the bias term and *s* is the activation function for which we used rectified linear function *max*(0, *x*). Similarly, decoding function can be represented as: *z* = *s* (*W*_2_*y* + *b*_2_), where we constrained it with tied weight *W*_1_ = *W*^*T*^ and *z* is the reconstructed data which is supposed to be close to input *x*. Squared error $\tfrac{1}{2}\parallel x-z{\parallel }^{2}$ is applied as loss function to optimize the network. The hypothesis is that the latent representation can capture the main factors of variation in the data. Comparing with another popular unsupervised feature learning method, the principle component analysis (PCA), the activation function enables the network to capture non-linear factors of data variation, especially when multiple encoders and decoders are stacked to form a SAE. To fully train the network, we applied greedy layer-wise training^36^ approach where every hidden layer was trained separately.

#### Supervised Fine-tuning

After pre-training, the first three layers of a DNN were initialized with the parameters of encoders from pre-trained SAE followed by a softmax output layer. At first, we trained the output layer independently while fixing the parameters of first 3 layers. Then we fine-tuned the whole network as Multilayer Perceptron (MLP) with subject labels for criterion. The network outputs the probabilities of a subject belonging to each class and the class with highest probability determines the output label of the subject. If we use *x*^*i*^, *y*^*i*^ to represent the input feature vector and label of the *i*_*th*_ sample, respectively, the loss function based on cross entropy can be displayed as:

1 $$\quad \quad \quad \quad \quad \quad \quad \quad \quad \quad \quad \quad \quad \quad H(i)=-\frac{1}{N}\sum _{i\mathrm{=1}}^{N}\sum _{j\mathrm{=1}}^{2}\mathrm{[1\{}{y}^{i}=j\}log(h{({x}^{i})}_{j}]$$

where *N* is the number of input samples, *j* represents the class of samples, and *h* represents the network function.

#### Optimization of Network

Training of the network was performed via back propagation with the Adam algorithm^37^. It is a first-order gradient-based optimization algorithm which has been proven to be computationally efficient and appropriate for training deep neural networks. During the training stage, the training set was randomly split into mini batches^38^ where each split contains 50 samples in this study. At every iteration, only a single mini batch was used for optimization. After every batch has been used once, the training set was reordered and randomly divided again so that each batch would have different samples in different epochs.

#### Dropout

In order to prevent the deep neural network from overfitting, regularization is necessary to reduce its generalization error. In this study, we used dropout^39^ to learn more robust features and prevent overfitting. In the dropout layer, some units were randomly dropped, providing a way to combine many different neural networks. In this study, we inserted dropout layers after every hidden layer. In each iteration of training stage, only half of hidden units were randomly selected to feed the results to the next layer, while in the testing stage all hidden units were kept to perform the classification. By avoiding training all hidden units on every training sample, this regularization technique not only prevented complex co-adaptations on training data and decrease overfitting, but also reduced the amount of computation and improved training speed.

#### Early Stopping

Another approach we used to prevent overfitting is early stopping. Because deep architectures were trained with iterative back propagation, the networks were prone to be more adaptive to the training data after every epoch. At a certain point, improving the network’s fit to the training set is likely to decrease generalization accuracy. In order to terminate the optimization algorithm before over-fitting, early stopping was used to provide guidance for how many iterations are needed. In the cross validation experiment, after dividing the data set into training and testing, we further split the training samples into a training set and a validation set. The networks were trained only with data in the former training set, while samples in the latter validation set were used to determine when to stop the algorithm: while the network has the highest generalization accuracy for validation set. In actual training, we stopped the optimization if the validation accuracy had ceased to increase for 50 epochs.

#### Ensemble Classifiers

Although early stopping has proven to be useful in most deep learning problems, relatively small data set limited the number of samples we could use for validation. And a small validation set may not able to represent the whole data set resulting in a biased network. Therefore, we resorted to ensemble multiple classifiers to perform more stable and robust classification. Instead of selecting a single validation set, we randomly divided the training set into 10 sets and used them to train 10 different networks to ‘vote’ for the classification. At the training stage, for network *i*, set *i* would be used for validation while the rest 9 sets were used for training. At the testing stage, the test samples were fed into all these networks resulting in 10 sets of probabilities. For each sample, the probabilities from 10 networks were added and the class with highest probability was the classification result of this sample. Although the performance of ensemble classifiers may not be greater than a single classifier on every occasion, the ensemble strategy can statistically improve the classification accuracy as well as the robustness and the stability of the classifier.

#### Ensemble Classifier Probability Distribution

The output of the DNN for each individual image is a pair of probability values representing the probabilities of the given input subject image features (or image pair features for multimodal images) as belonging to one of the two classes on which the DNN was trained. This probability score for belonging to the disease positive (AD) class can be interpreted as a disease severity staging score, since value of 1 represents the highest probability of being from the AD class, and 0 represents highest probability of being from the disease negative (NC) class.

### Classifier Validation Experiment Setup

To validate the discriminant ability of proposed network, two kinds of binary classification experiments were performed. First, we performed discrimination between sMCI and pMCI to compare our results on this experiment directly with the published state-of-the-art methods^18,20,21,28,40–46^. Since the published literature typically used only baseline images, we also used a single baseline image for each of the 409 sMCI subjects. Hence, the number of sMCI images is the same as the number of sMCI subjects. For the 217 pMCI subjects, their earliest image within 3 years before conversion was selected. The data samples were randomly divided into 10 sets. For each iteration, 1 set was used for testing while the rest sets were all used for training. Therefore, all subjects were used for testing exactly once.

One potential issue with respect to the sMCI class is that some of these individuals may progress to AD or other dementias in the future and if some of these individuals convert to probable AD in the future, these earlier timepoints would become part of the pMCI group, whereas some other individuals may revert back to NC. Hence, although the sMCI vs. pMCI experiment is commonly used to assess classifier performance in recent studies, the classification of sMCI subjects may not be entirely accurate due to the potential uncertainty in the clinical diagnosis of the sMCI class. Therefore, we performed additional experiments that involved classifying individuals with known future progression to AD, namely the pNC, pMCI and sAD classes, denoted as the dementia positive class, against those that are stable normal controls (sNC), denoted as the dementia negative class.

We investigated the performance of the classifier by using various combinations of samples during training phase. At the first level, the classifier was trained soley on samples from the sNC subjects (the dementia negative class) and the sAD subjects (the dementia positive class). At the next level, the dementia positive class was enriched with pMCI subjects’ images that represent an earlier stage in the evolution of AD. In the last level, the positive class was further enriched with adding pNC subjects’ images representing an even earlier stage in the evolution of AD. For each level, the classifier training followed the standard 10-fold cross validation procedure (90% of data samples used for training and 10% of data used for testing in each iteration). The groups not used for training, if any, were utilized in the testing group. In these experiments, allocation into training or testing was done on the level of subjects, not images. If a subject was allocated into the training group, all the available baseline and longitudinal images for this subject would be used for training. Otherwise, all the available images of a subject would be used for testing.

Sensitivity of the classifier is defined as the number of positive class images that are correctly classified, which in this case is the classification of the test subset of pNC, pMCI and sAD images as the positive class. Specificity of the classifier is the number of negative class images (the sNC class) that are correctly classified as sNC. Accuracy of the classifier is the fraction of images from both the positive and the negative classes that are correctly classified.

The proposed deep neural network (DNN) was built with Tensorflow^47^, an open source deep learning toolbox provided by Google. For all the experiments, the number of nodes in each layer was predefined as shown in Fig. 2 and the learning rate was set as 10^−4^. The deep network parameter space is very large, with a large range of choices from which to sample i.e. number of layers and number of nodes, testing all the possible parameter combinations exhaustively is computationally unrealistic. Instead of doing parameter selection for each of the 10-fold experiments, the parameters were selected based on the results of the first fold experiment.

## Results

### Discrimination between Stable and Progressive MCI (sMCI vs pMCI)

We conducted the sMCI vs. pMCI experiment to be able to compare the classification accuracy of our proposed novel method with published and comparable state-of-the-art methods^18,20,21,28,40–46^. The FDG-PET image and MRI image acquired at a single time point for each subject were used for the 10-fold cross validation experiment. For sMCI subjects, the images acquired at the first time to visit, while for pMCI subjects, the images acquired at the earliest time point within 3 years before conversion were used. Results of this experiment and comparable results from published methods are shown in Table 2. These results reveal an accuracy of 82.9% for our MMDNN method over 626 subjects and both specificity (83.8%) and sensitivity (79.7%) are high. The results for single modality DNN are also found to improve upon the state-of-art. These results suggest that our proposed MMDNN network is promising for applications requiring classification between sMCI and pMCI individuals for the single modality T1-MRI and FDG-PET or the multimodal (T1-MRI and FDG-PET combined) neuroimaging approach.

**Table 2:** Classification performance for the sMCI vs. pMCI task: Accuracy (%), Sensitivity (%), and Specificity (%) of the proposed network compared with the published state-of-the-art methods for the task of discriminating between sMCI and pMCI subjects.

Method	Modality	Year to conversion	#Subjects	Accuracy	Sensitivity	Specificity
Young *et al*.^18^	MRI	0–3	143	64.3	53.2	69.8
Liu *et al*.^40^	MRI	0–3	234	68.8	64.29	74.07
Suk *et al*.^28^	MRI	unknown	204	72.42	36.7	90.98
Cheng *et al*.^41^	MRI	0–2	99	73.4	74.3	72.1
Zhu *et al*.^42^	MRI	0–1.5	99	71.8	48.0	92.8
Huang *et al*.^46^	longitudinal MRI	0–3	131	79.4	86.5	78.2
**Proposed**	MRI	0–3	**626**	**75**.**44** (7.74)	73.27 (7.58)	76.19 (8.35)
Young *et al*.^18^	PET	0–3	143	65.0	66.0	64.6
Liu *et al*.^40^	PET	0–3	234	68.8	57.14	82.41
Suk *et al*.^28^	PET	unknown	204	70.75	25.45	96.55
Cheng *et al*.^41^	PET	0–2	99	71.6	76.4	67.9
Zhu *et al*.^42^	PET	0–1.5	99	71.2	47.4	93.0
**Proposed**	PET	0–3	**626**	**81**.**53** (7.42)	78.20 (7.72)	82.47 (9.30)
Young *et al*.^18^	MRI + PET + APOE	0–3	143	69.9	78.7	65.6
Liu *et al*.^40^	PET + MRI	0–3	234	73.5	76.19	70.37
Suk *et al*.^28^	PET + MRI	unknown	204	75.92	48.04	95.23
Cheng *et al*.^41^	PET + MRI + CSF	0–2	99	79.4	84.5	72.7
Zhu *et al*.^42^	MRI + PET	0–1.5	99	72.4	49.1	94.6
Moradi *et al*.^20^	MRI + Age + cognitive measure	0–3	264	82	87	74
Xu *et al*.^43^	MRI + PET + florbetapir PET	0–3	110	77.8	74.1	81.5
Zhang *et al*.^44^	longitudinal MRI + PET	0–2	88	78.4	79.0	78.0
An *et al*.^45^	MRI + SNP	0–2	362	80.8	71.5	85.4
Korolev *et al*.^21^	MRI + Plasma + cognitive measure	0–3	259	80.0	83.0	76.0
**Proposed**	PET + MRI	0–3	**626**	**82**.**93** (7.25)	79.69 (8.37)	83.84 (6.37)

‘PET’ in this table represents FDG-PET neuroimaging. Our proposed approach using deep neural networks was performed using a single FDG-PET image and a single T1-MRI acquired from each of 409 sMCI subjects and 217 pMCI subjects (total 626 subjects) and the average (standard-deviation) of the accuracy, sensitivity and specificity of classifier performance are reported.

### Discrimination between disease negative (sNC) and disease positive (the pNC, pMCI, sAD) classes

The classifier was trained to discriminate the negative class (sNC) from the disease positive class (pNC, pMCI, sAD) using three different enrichments for the positive class samples, namely training with the positive class containing only sAD, or, pMCI and sAD, or, pNC and pMCI and sAD samples. Each subject was used for testing at least once in the 10-fold cross validation experiments. In each fold of the experiment, images of the same subject acquired at different time points were either all used for training or all used for testing to ensure the independence of training and testing at all times, as further detailed in the Classifier Validation Experiment Setup Section.

The classification result of these experiments are shown as Table 3. The DNN based on FDG-PET neuroimaging features (accuracy 85.9%) performs better than the DNN based on T1-MRI (accuracy 82.5%) neuroimaging features, and the combined MMDNN outperforms each of the single modality DNNs (accuracy 86.4%). As the positive class is enriched with samples from the pMCI and then further with the pNC samples, there is an increase in the sensitivity (correctly classified members of the dementia positive class i. e. pNC, pMCI and sAD). Since some of the early stage patterns of AD represented in pMCI and pNC may overlap the sNC group, there is a slight decrease in specificity, but overall an increase in accuracy.

**Table 3:** Accuracy (%), sensitivity (%) and specificity (%) of each modality and the multimodal combination using different training sets for the classification of AD pathology (discrimination of pNC, pMCI and sAD from sNC).

	FDG-PET (Metabolism)			T1-MRI (Volume)			Multimodal (Metabolism + Volume)		
	Acc.	Sens.	Spec.	Acc.	Sens.	Spec.	Acc.	Sens.	Spec.
**Training Set**									
sNC vs. sAD	84.5 (1.4)	79.9 (1.6)	91.9 (6.9)	81.9 (1.2)	75.5 (1.3)	92.3 (4.9)	84.6 (1.5)	80.2 (2.0)	91.8 (6.8)
sNC vs. (pMCI and sAD)	85.5 (2.8)	85.0 (2.9)	86.2 (10.1)	82.8 (3.4)	79.8 (4.1)	87.7 (6.3)	86.0 (2.5)	85.7 (3.2)	86.5 (8.6)
sNC vs. (pNC, pMCI and sAD)	85.9 (4.8)	85.6 (3.8)	86.3 (7.8)	82.5 (5.2)	80.2 (7.6)	86.1 (7.6)	86.4 (4.7)	86.5 (5.2)	86.3 (8.6)

The numbers in each cell are the average value and standard deviation from the 10-fold cross validation experiments. Sensitivity is the fraction of correctly classified pNC, pMCI and sAD images, specificity is the fraction of correctly classified sNC images and accuracy is correctly classified images taken together.

**Figure 3:**
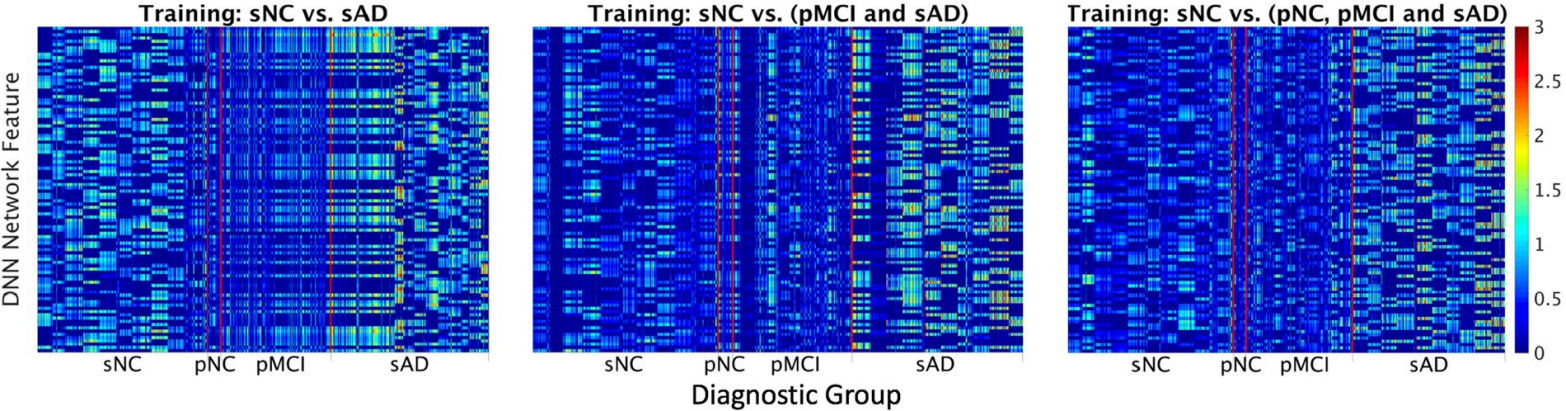
Features extracted from the input data by the deep neural network at the penultimate layer that are fed to the output layer for classification. From left to right the training set is sNC vs sAD, sNC vs (pMCI + sAD), and sNC vs (pNC + pMCI + sAD) respectively. The y axis represents the units of the second from last layer, while the x axis denotes the different data groups. The vertical red lines are added to enhance visual distinction between the boundaries of each group. This figure shows that while it is difficult to provide an interpretation of the features found by the deep neural network from the input neuroimaging features, the patterns as distilled by the deep learning network from the sNC, pNC, pMCI and sAD images are distinct with more uniformity within each class as compared to across classes.

The features extracted by the deep neural network are displayed in Fig. 3. Although difficult to interpret as these are extracted from multiple nonlinear transformations of data, they show that the patterns for the different classes appear to be distinct, whereas patterns within each class appear to be relatively similar.

### Classification performance of pNC and pMCI as function of time (years) to conversion

**Figure 4:**
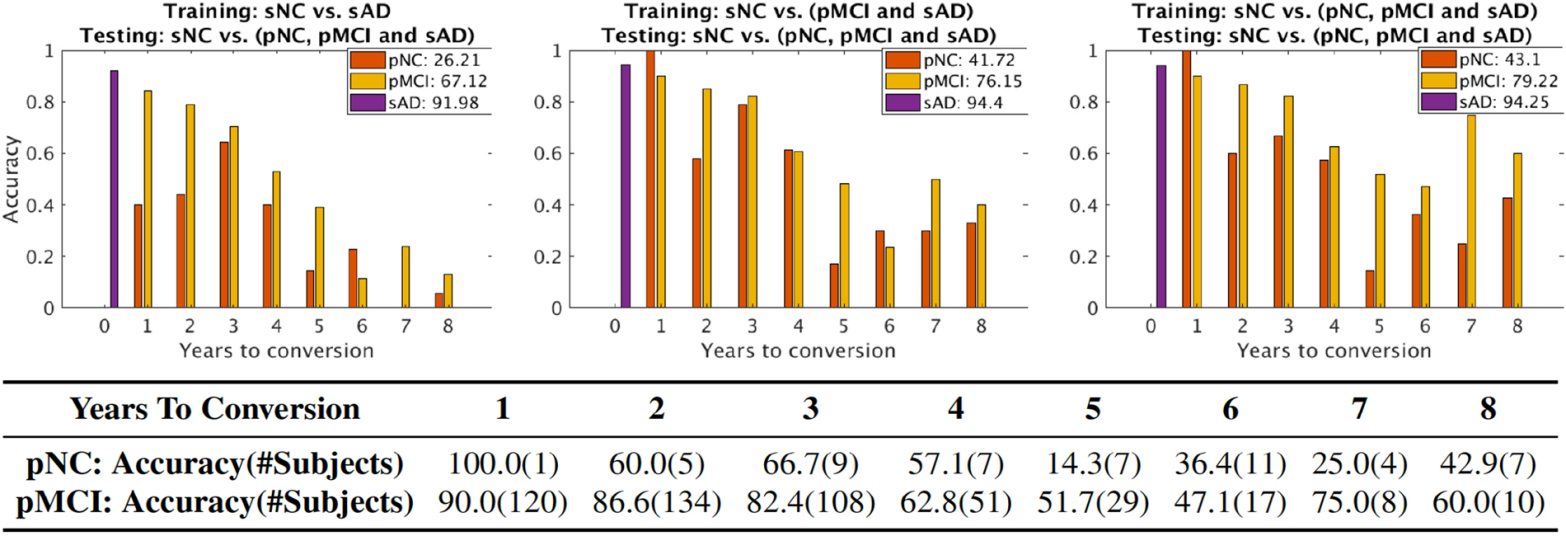
Accuracy of correctly identifying prodromal AD for the pNC and pMCI subjects as function of (years) to conversion. The top row shows the effect of enriching the training set of the dementia positive class, with sAD (top row, left panel), pMCI and sAD (top row, middle panel) and pNC and pMCI and sAD (top row, right panel). The y axis represents accuracy of classification, while x axis shows time (years) to AD conversion. The x axis value ‘0’ indicates subjects with current clinical diagnosis of probable AD (sAD subjects). The number in legend is the classification accuracy taken over all time points for each group. The table in the second row shows the numeric accuracy value for pNC and pMCI subjects at different time (years) to conversion to AD corresponding to the top right panel (MMDNN with pNC, pMCI and sAD for training the dementia positive class).

We analyzed the accuracy of classification of pNC and pMCI as a function of the time (years) to conversion and the numbers of subjects available for the MMDNN classifier. These results are shown in Fig. 4 for each of the three training scenarios with progressive enrichment of the positive class. As the positive class training set of sAD (top row, left panel) is enriched with samples from pMCI (top row, middle panel) and with pNC and pMCI samples (top row, right panel), the accuracy of detection of the pMCI and pNC class increases, as well as an increase in accuracy for identifying AD in pNC and pMCI earlier. The numerical values of classifier performance for the pNC, pMCI and sAD enriched positive class (top row, third panel on the right) are provided in the table in the second row of this figure.

The MMDNN classifier accuracy in identifying pMCI individuals with future conversion to AD was 90%, 86.6% and 82.4%, for years 1, 2, and 3 away to conversion. The accuracy for all the years taken together for pMCI classification was 79.22%, and 86.4% total for conversion within 1–3 years. The neuroimaging scans farther away from conversion are likely more challenging to classify correctly leading to overall lowered accuracy. The classification accuracy for sAD group, i. e, those images associated with a clinical diagnosis of AD, is 94.25%. The accuracy for correctly classifying all pNC images is 41.1% with higher numbers of 100%, 60.0% and 66.7% for years 1, 2 and 3 from conversion to clinical diagnosis of probable AD.

### Classification Probability score distribution

**Figure 5:**
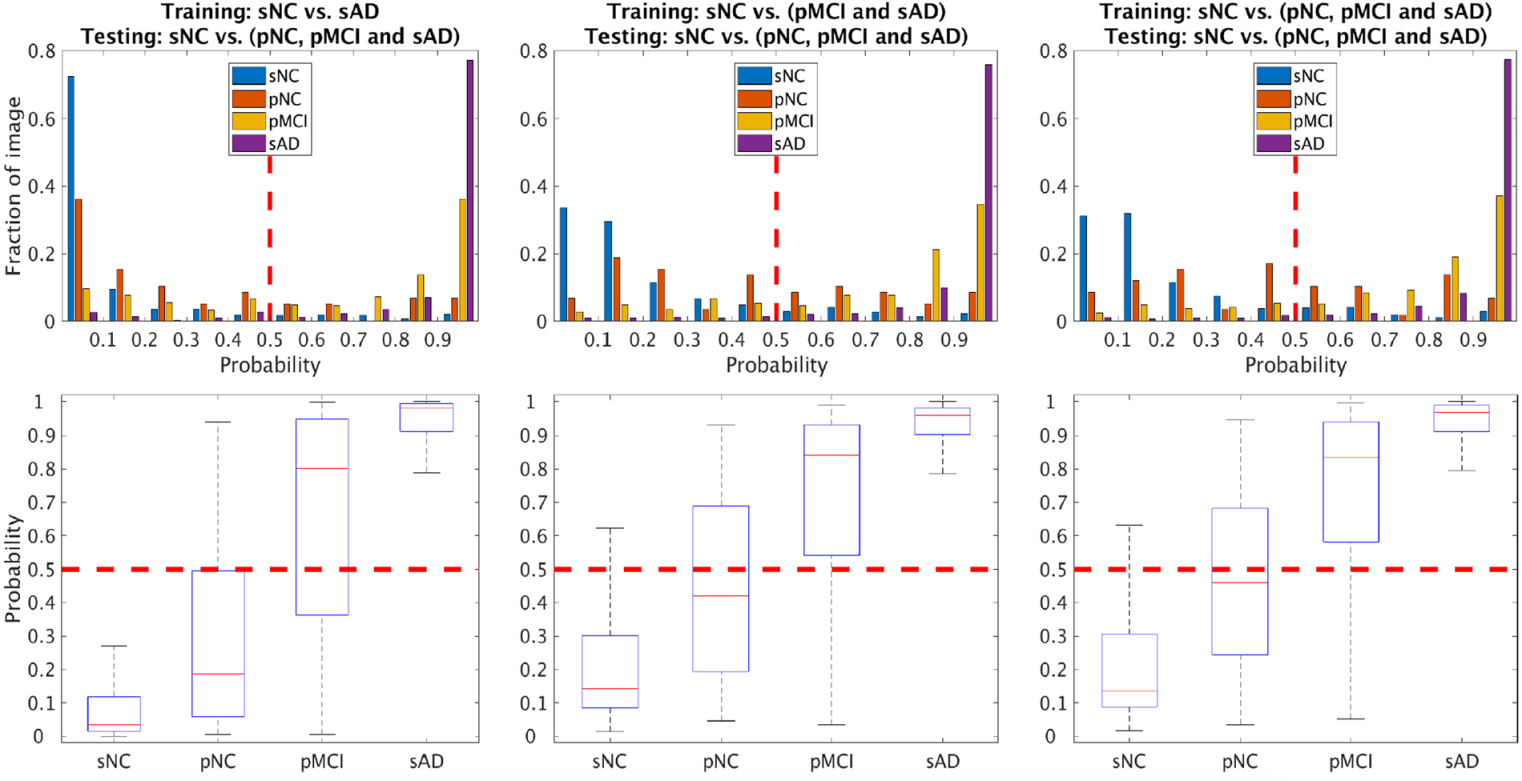
Multimodal classification probability distribution of different training sets. From left to right the training set is sAD, sAD and pMCI, sAD, pMCI and pNC versus sNC respectively. The y axis represents fraction of images, while x axis denotes the probability of class sAD, where 0 represents high likelihood of being from the sNC pattern, and 1 represents high likelihood of being from the sAD pattern.

The probability score output by the MMDNN trained with the dementia negative (sNC) class and the three enrichment choices for the dementia positive class (namely, sAD, pMCI + sAD, and pNC + pMCI + sAD) class samples is visualized as histograms in the top row of Fig. 5. The fraction of images of each class is shown on the y axis, along with classifier probability score shown on the x axis. This distribution shows how the sNC, pNC, pMCI and sAD classes are scored by the classifier for their probability of being from the dementia positive class. Further, the bottom row of Fig. 5 shows aggregate values of the probability score with respect to each class with a box plot. As the training set for the dementia positive class is enriched with samples from pMCI and then additionally, pNC class, the probability score for these classes is shown to increase. Overall, the distribution generated by the MMDNN leads to good separation between the classes, and the threshold choice of 0.5 (highest class probability assignment) is visually shown to provide good classification between the classes.

## Discussion

In this paper, we have proposed a novel deep neural network (DNN) based method that utilizes multi-scale and multi-modal information (MMDNN) combining metabolism (FDG-PET) and regional volume (T1-MRI) for the discrimination of AD, with a focus on assessing classification accuracy in those pNC and pMCI subjects with known future conversion to probable AD. In accordance with scale-space theory, our incorporation of multiscale approach was intended to capture the discriminant signals at multiple scales, and avoid apriori assumption of the scale at which the discriminant signals may reside.

The comparison between our novel proposed MMDNN method and state-of-the-art methods for the sMCI vs. pMCI classification task is shown in Table 2. Although the data used for the cited studies are not identical, they all come from the ADNI database and have comparable image acquisition and preprocessing procedures. One of the strengths of our work is that we have analyzed all the available ADNI sMCI and pMCI subjects having both MRI and FDG-PET neuroimages at the time of preparation of this manuscript. When using only the T1-MRI modality, our method has better accuracy than most methods expect Huang *et al*.’s^46^. However, they used a longitudinal method with multiple MRI images acquired from different time points for the classification of each subject, whereas we classify each image separately, an approach consistent with the other published cross-sectional methods. For single modality-based classifiers using only FDG-PET, our method outperforms the published methods by a significant margin as shown in Table 2. Extension of our DNN for utilization of longitudinal timepoints for single subject classification is a direction for future work, and we anticipate that adding longitudinal measures explicitly could further improve the classifier performance.

When using multiple modalities for sMCI vs. pMCI classification, our MMDNN approach has the best performance specially compared with the methods that also used the same T1-MRI and FDG-PET modalities. The study of Chen *et al*.^41^ performed domain transfer learning to exploit the auxiliary domain data (sAD/sNC subjects) to improve the classification whereas our proposed MMDNN method’s performance was better even though we did not utilize domain transfer learning in our sMCI vs. pMCI classification task.

Further, we performed experiments to detect prodromal AD by training the MMDNN classifier with samples from the dementia positive class namely the pNC, pMCI and sAD subjects. The accuracy of correctly classifying pNC and pMCI subjects as having patterns indicative of AD improved when the classifier training included pMCI and pNC images, as displayed in Table 3. Further, comparison of the DNN results for T1-MRI and FDG-PET classifiers as shown in Table 3 indicates that the sensitivity of detection of prodromal AD is higher with FDG-PET neuroimaging features as compared to T1-MRI neuroimaging features. This finding is consistent with previous studies^18,28,40,41^ and could indicate support for the hypothesis that alterations in metabolism may precede changes in structure, and further, the altered metabolism measures could be detected with FDG-PET earlier than the detection of structural changes with T1-MRI.

Analysis of the accuracy of classifying prodromal AD i. e. detecting patterns corresponding to AD in pNC and pMCI individuals as function of time (years) to conversion is shown in Fig. 4. As the training set was enriched with samples from the pNC and the pMCI groups, the accuracy of detection of prodromal AD also increased. The MMDNN classifier delivered high accuracy upto three years prior to conversion and then performance was reduced for the timepoints 4–8 years prior to conversion. The number of subjects in 1–3 years before conversion are large (over 100 each), and there is also reduced numbers of available subject numbers 4–8 years away from conversion. The reduced sample for timepoints farther away from conversion to AD could potentially increase classification uncertainty. With more neuroimaging data corresponding to timepoints farther from conversion to AD becoming available, models such as the MMDNN proposed here could provide better classification performance for the earlier detection of prodromal AD.

The probability score output from the DNN is visualized in Fig. 5. The probability score is highest for the sAD class, and lowest for the sNC class, being the two extreme ends of the spectrum for the classifier. The probability score for the pNC and pMCI subjects is in between, and higher for pMCI than pNC generally in line with the expectation of progressive alterations detected with neuroimaging for subjects further along the disease trajectory. Further analysis of the classifier probability score could be an interesting avenue to develop a surrogate staging score for disease severity.

Despite the remarkable ability of DNN to discover patterns that may not be apparent on human visual examination, one major disadvantage of the DNN framework is that as a result of multiple non-linear transformations between the input in generating the output, it is not readily possible to map the output classification probability back to neuroimaging patterns in the input neuroimaging data that give rise to this output. The visualization of the output of the penultimate layer in the DNN for individual subject images is shown in Fig. 3 and except for observing a qualitative difference between the features of different classes, it is not possible to relate these to neuroimaging features from specific locations in the brain at the current time. Understanding how to provide pathophysiologically meaningful interpretation of the features extracted by the DNN for classificaion remains an unsolved problem and is an important future research direction.

A small number of subjects are awarded a probability score inconsistent with their clinical diagnosis. One of the main requirements of training DNNs are large quantities of well-characterized data^25^. It is therefore possible that as more comprehensive and homogeneous training databases are developed and become available for learning, the accuracy numbers may increase and these outliers will be reduced. It is also possible that there may be some uncertainty in the available clinical diagnosis. Despite the limitations, our findings indicate that the DNN framework has considerable potential in learning the AD-related patterns for promising future applications in adding to the toolbox of clinical AD diagnosis.

## Conclusion

In summary, we have proposed a novel deep neural network to identify individuals at risk of developing Alzheimer’s disease. Our multi-scale and multi-modal deep neural network (MMDNN) was designed to incorporate multiple scales of information from multiple regions in the gray matter of the brain taken from multiple modalities (T1-MRI and FDG-PET). First we demonstrated the discriminant ability of the proposed MMDNN approach by comparing with state-of-the-art methods on the task of discriminating between sMCI vs. pMCI individuals. Then we trained the classifier to distinguish subjects on trajectory towards clinical diagnosis of probable AD (i. e. the pNC, pMCI subjects). We observed the performance of MMDNN classifier built with a combination of FDG-PET and structural MRI images was better than those built using either structural MRI or FDG-PET neuroimaging scans alone. Further the classifier trained with the combined sample of pNC, pMCI and sAD was found to yield the highest overall classification accuracy of 82.4% accuracy in the identifying the individuals with mild cognitive impairment (MCI) who will convert to AD at 3 years prior to conversion (86.4% combined accuracy for conversion within 1–3 years), a 94.23% sensitivity in classifying individuals with clinical diagnosis of probable AD, and a 86.3% specificity in classifying non-demented controls. These results suggest that deep neural network classifiers may be useful as a potential tool for providing evidence in support of the clinical diagnosis of probable AD.
